# Association Between Postoperative Day 7 Hypoalbuminemia and Mortality After Surgery: A Single-Center Retrospective Landmark Cohort Study

**DOI:** 10.3390/jcm15197742

**Published:** 2026-10-08

**Authors:** Tatsuro Yokoyama, Koshiro Kikkawa, Daiki Sakaguchi, Hiroha Shibata, Ryohei Ito

**Affiliations:** 1 Department of Intensive Care Medicine, Ogaki Municipal Hospital, 4-86 Minaminokawa, Ogaki 503-8502, Gifu, Japan; 2 Department of Anesthesiology, Ogaki Municipal Hospital, 4-86 Minaminokawa, Ogaki 503-8502, Gifu, Japan; kikkawa0330@gmail.com (K.K.); d.sakaguchi1218@gmail.com (D.S.); hiroha0213@yahoo.co.jp (H.S.); ryochingnet@gmail.com (R.I.)

**Keywords:** hypoalbuminemia, frailty, effect modification, postoperative mortality, surgical urgency, landmark analysis

## Abstract

**Background/Objectives**: We evaluated postoperative day 7 serum albumin for its association with mortality after surgery and whether it varied by frailty or surgical urgency. **Methods**: This single-center landmark cohort study was conducted at an 817-bed tertiary hospital (April 2018–March 2025). Follow-up began on day 7 to reduce immortal-time bias. Of 41,506 surgical episodes, 18,296 met the landmark and 14,992 (82%) had day 7 albumin measured. Hypoalbuminemia was <3.0 g/dL. We assessed 365-day mortality by landmark Cox regression. We compared discrimination with the preoperative Surgical Outcome Risk Tool (SORT) and American Society of Anesthesiologists (ASA) physical status, added day 7 albumin to each, and tested effect modification by frailty and urgency. **Results**: Adding day 7 albumin improved discrimination of post-landmark in-hospital mortality (area under the curve 0.638 to 0.769). When added to SORT, discrimination rose from 0.662 to 0.803, and when added to ASA physical status, from 0.700 to 0.837. Temporal internal validation yielded similar AUCs (33 deaths). Day 7 hypoalbuminemia was associated with higher 365-day mortality (adjusted hazard ratio 3.43; 95% CI, 2.95–3.98), attenuating to 1.89 (95% CI, 1.60–2.23) after adjustment for preoperative albumin. No clear interaction with frailty emerged; an exploratory inverse interaction with emergency surgery was observed (odds ratio, 0.30; 95% CI, 0.13–0.68). **Conclusions**: Day 7 hypoalbuminemia, possibly reflecting the overall postoperative course, was associated with short- and long-term mortality and improved discrimination beyond routine and preoperative models. Effect modification was limited and exploratory. These hypothesis-generating findings require external validation and prospective assessment of clinical utility.

## 1. Introduction

Serum albumin is one of the most consistent laboratory markers associated with surgical outcomes. However, operative risk is typically assessed preoperatively using prediction tools. A postoperative measurement may provide greater prognostic value because it reflects the patient’s physiological response to the actual surgical insult rather than the risk anticipated before surgery. Whether postoperative day 7 hypoalbuminemia adds prognostic information beyond established preoperative risk tools and whether its association varies by frailty or surgical urgency remain unclear.

Lower preoperative albumin is associated with postoperative mortality and morbidity across large cohort studies and meta-analyses, including cancer surgery [1,2,3,4,5]. Postoperative albumin dynamics may provide additional information: serum albumin falls within hours of surgery in proportion to operative severity [6], predicts complications earlier than C-reactive protein [7], and remains prognostic even when preoperative albumin is normal [8]. This decline reflects the inflammatory response rather than nutritional status, because albumin is a negative acute-phase protein [9], which may explain why supplementation has not consistently improved postoperative outcomes [10]. Likewise, the C-reactive protein-to-albumin ratio has been associated with perioperative mortality [11].

Despite these findings, three gaps remain. First, postoperative day 7 albumin has rarely been evaluated using a landmark design that reduces immortal-time bias by restricting analysis to patients alive at a fixed time point [12,13]. Second, although frailty and surgical urgency are well-established determinants of postoperative mortality [14,15] and a database-derived index combining them outperformed either alone [16], previous studies have focused on their prognostic value rather than whether either modifies the association between postoperative albumin and mortality, with interaction potentially differing on multiplicative and additive scales [17,18,19]. Third, the commonly used preoperative tools, the Surgical Outcome Risk Tool (SORT) and American Society of Anesthesiologists (ASA) physical status, are fixed before surgery [20], and few studies have compared their prognostic performance with postoperative biomarkers.

We hypothesized that postoperative day 7 albumin, by reflecting the realized rather than anticipated surgical insult, including any early complications, would carry prognostic information not captured by preoperative tools. We therefore had three objectives. First, to estimate the association between day 7 hypoalbuminemia and postoperative mortality using a landmark cohort design. Second, to assess whether day 7 albumin adds discriminative information to the established preoperative tools, the SORT and ASA physical status, rather than whether it outperforms them. Third, as a secondary and exploratory aim, to examine whether frailty or surgical urgency modifies this association.

## 2. Materials and Methods

### 2.1. Study Design and Data Source

This single-center retrospective cohort study at Ogaki Municipal Hospital, an 817-bed tertiary care center in Japan, used the hospital’s integrated electronic health records (surgical, anesthetic, laboratory, pharmacy, discharge, and mortality). Index surgical procedures performed from 1 April 2018 through 31 March 2025 were eligible, with follow-up data available through August 2026. The statistical analysis plan was finalized before outcome analyses. Reporting followed the Strengthening the Reporting of Observational Studies in Epidemiology (STROBE) guideline (STROBE checklist).

### 2.2. Study Population

#### 2.2.1. Inclusion Criteria

Adults (≥18 years) admitted for a surgical procedure with a corresponding anesthetic record during the study period were eligible and were identified by linking surgical and hospital discharge records.

#### 2.2.2. Exclusion Criteria

**Figure 1:**
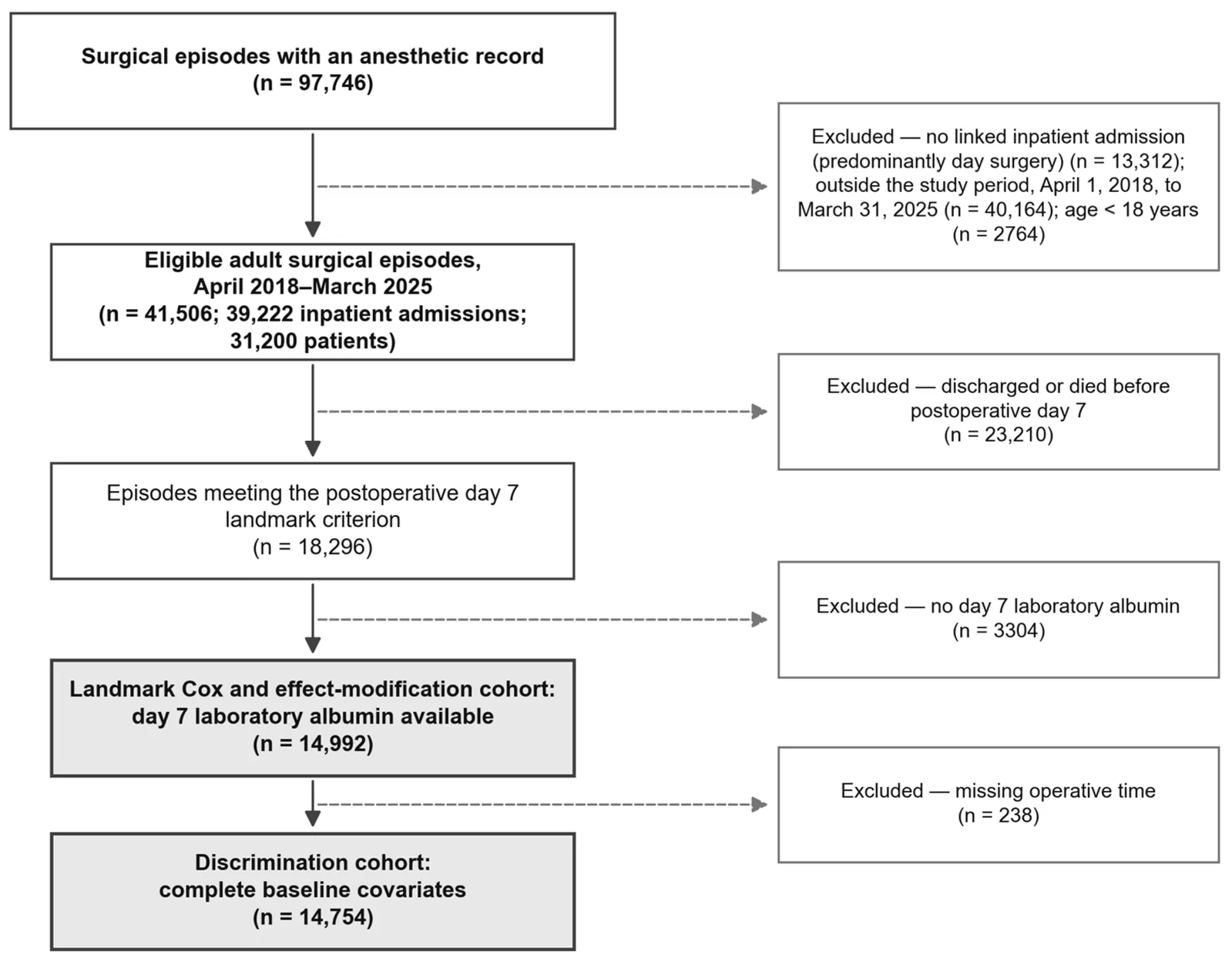
Cohort flow diagram. Surgical episodes with an anesthetic record were screened for eligibility. Episodes without a linked inpatient admission, which were predominantly day surgery, episodes outside the study period, and episodes in patients aged < 18 years were excluded, leaving 41,506 eligible surgical episodes from 39,222 inpatient admissions among 31,200 patients. Episodes meeting the postoperative day 7 landmark criterion and having a day 7 laboratory albumin measurement formed the landmark Cox and effect-modification cohort. Episodes with complete baseline covariates formed the discrimination cohort. Each qualifying operation was treated as a separate study episode, so an inpatient admission with more than one operation contributes more than one episode.

Patients aged <18 years were excluded, as were surgical episodes without a linked inpatient admission, which were predominantly day-surgery procedures performed under local or regional anesthesia and for which inpatient outcome ascertainment was not possible (Figure 1).

In the primary analysis, each qualifying operation was treated as a separate study episode, with 41,506 episodes arising from 39,222 inpatient admissions. To account for potential within-patient correlation, a sensitivity analysis was performed using a single episode per patient, selecting the emergency episode where present and otherwise the episode with the longest operating-room time.

### 2.3. Exposure and Landmark

The exposure was postoperative day 7 hypoalbuminemia, defined as serum albumin < 3.0 g/dL from the measurement closest to postoperative day 7 (obtained on days 4–7 so that it preceded the landmark). Day 7 was chosen because it is late enough for the metabolic response to surgery to be reflected in serum albumin, yet early enough to precede most in-hospital deaths and discharge decisions. Because albumin was not measured daily, the value closest to day 7 was used; 73.5% of measurements fell on day 7 itself and the remainder on days 4 to 6, and the proportion below 3.0 g/dL varied little across those days (50.4% to 56.7%; Table S9). To minimize immortal-time bias [12,13], analyses were restricted to episodes in which the patient was still hospitalized on postoperative day 7 and thus eligible for a day 7 measurement; anchoring follow-up at a fixed postoperative day, rather than at the time the sample was drawn, prevents an episode from being counted as exposed over a period in which its exposure was not yet defined. Of the 18,296 episodes meeting this landmark criterion, 14,992 had a day 7 albumin measurement, and Table S12 compares the 3304 without one with episodes in which albumin was measured. Albumin values from nutrition care forms were excluded because they could reflect carried-forward preoperative measurements (Appendix A). The threshold of 3.0 g/dL was chosen to identify severe hypoalbuminemia rather than any value below the laboratory reference range; the conventional definition in surgical cohorts is 3.5 g/dL [2,3,8], and a threshold of 3.4 g/dL has been derived empirically in a mixed surgical population [2]. Results at 3.5 g/dL are reported as a post hoc sensitivity analysis (Table S10), with the reclassification between the two thresholds in Table S11. Albumin was modeled as binary (<3.0 vs. ≥3.0 g/dL) for interaction analyses and as continuous for discrimination analyses to avoid information loss from dichotomization.

### 2.4. Outcomes

The primary outcome was all-cause mortality, ascertained from the hospital death registry, which records in-hospital deaths and those occurring outside the hospital that are reported to the institution, with censoring at the last documented contact with the institution. The observation window extended beyond 365 days after the latest index surgery for every episode; therefore, incomplete ascertainment reflects loss of contact with the institution rather than truncation at the end of the study period. Two primary analyses were prespecified: first, the association between day 7 hypoalbuminemia and mortality at 365 days after surgery; and second, the incremental discriminative value of day 7 albumin over a routine postoperative baseline model, together with a head-to-head comparison against established preoperative tools, for post-landmark in-hospital mortality, a fully observed endpoint that allowed comparison within the same episodes. Adding day 7 albumin to each preoperative tool was not prespecified and is reported post hoc (Table S16). Mortality at 30 and 90 days comprised secondary endpoints. Effect modification by frailty and surgical urgency was a prespecified secondary exploratory analysis of post-landmark in-hospital mortality.

### 2.5. Covariates and Effect Modifiers

The two prespecified effect modifiers were frailty and surgical urgency. Frailty was assessed with the Hospital Frailty Risk Score, dichotomized at ≥5 to separate the low-risk group from the intermediate- and high-risk groups defined by Gilbert et al. [14]. The score comprises the 109 ICD-10 diagnosis codes and weights reported in that derivation. The Charlson Comorbidity Index was derived with the ICD-10 coding algorithms of Quan et al. [21] and the updated weights validated across six countries [22], which update the original index of Charlson et al. [23]. Both scores were derived from diagnoses with an onset before the index surgery at our institution. Surgical urgency was classified as elective or emergency. Adjustment covariates were age, sex, and the Charlson Comorbidity Index (Table S1), and models for each effect modifier were additionally adjusted for the other modifier. The frailty score was analyzed as a binary variable at the prespecified threshold; a post hoc analysis using the three published bands is given in Table S19. Preoperative albumin, available within 30 days before surgery for 13,781 (91.9%) of the landmark cohort, was not included in the primary adjustment set, which had been prespecified before these values were identified; it is described, together with its change to day 7, in Table S14. Adjusting for it would change the question from the prognostic information carried by the day 7 value as observed at the landmark to the information added by the postoperative course beyond the preoperative value; models including it are therefore reported separately (Table S21). Because postoperative albumin infusion may raise measured serum albumin without reflecting endogenous production, infused albumin was not used to define the exposure, and its potential influence was evaluated in a prespecified sensitivity analysis.

### 2.6. Statistical Analysis

The association between day 7 hypoalbuminemia and 365-day mortality was evaluated with landmark Cox proportional hazards regression, adjusted for age, sex, and the Charlson Comorbidity Index, with censoring at the last documented contact or 365 days after surgery, whichever occurred earlier. The proportional hazards assumption was checked with scaled Schoenfeld residuals. Because approximately one in six patients contributed more than one episode, cluster-robust (sandwich) standard errors were estimated at the patient level. Discrimination for post-landmark in-hospital mortality was assessed using the area under the receiver operating characteristic curve (AUC), with Harrell’s bootstrap optimism correction based on episode-level resampling (a patient-level cluster bootstrap is given in Table S6) in a single common sample with all predictors available, using nested models: a routine baseline (age, surgical urgency, anesthetic type, and operative time), then sequential addition of day 7 albumin and day 7 C-reactive protein. C-reactive protein was modeled as a continuous linear term in mg/dL, without transformation or categorization; for the discrimination models, all continuous predictors were z-standardized, and the models were fitted with L2 regularization, whereas the Cox models used untransformed units. For head-to-head comparison, these models were evaluated against the preoperative SORT [20] and ASA physical status in the same sample, which required complete predictor data, a computable SORT probability, and a documented ASA status. In a post hoc analysis, day 7 albumin was added to each preoperative tool and to the two combined, and each tool was compared with the corresponding augmented model within a single complete-case sample containing every predictor that both models required (Table S16). All models predicted the same outcome; 95% confidence intervals (CIs) were bootstrap-derived. For temporal (out-of-time) validation, models were developed using 2018–2023 data and validated in the 2024 cohort. Calibration was assessed using the Brier score and the Hosmer–Lemeshow statistic with decile grouping and was shown graphically by decile of predicted risk (Table S17 and Figure S1); net benefit was explored by decision-curve analysis (Table S18); both were apparent (in-sample), and only the area under the curve was corrected for optimism. Mortality at 365 days was the primary horizon because it captures deaths after discharge that an in-hospital outcome cannot, and results at 30 and 90 days are reported alongside it. Because the primary adjustment set did not include procedure type, a post hoc model additionally adjusted for Major Diagnostic Category (Table S20). Because the primary adjustment set also excluded operative magnitude, the preoperative albumin concentration, postoperative inflammation, and frailty, a further post hoc series added each variable in turn and then all variables together. Each model is reported beside the primary adjustment set fitted in the same episodes so that a change in the estimate cannot be attributed to a change in sample (Table S21). The fall in albumin from the preoperative value to day 7 was also fitted as a continuous exposure in place of the day 7 threshold, with and without the preoperative value in the model.

Effect modification was assessed on both scales, because interaction estimates need not agree when baseline risks differ, and the additive scale is more directly relevant to absolute-risk differences when considering bedside targeting. Multiplicative interaction used the interaction odds ratio from a logistic model with a product term, adjusted for age, the Charlson Comorbidity Index, and the other modifier. Additive interaction used the relative excess risk due to interaction (RERI), with stratum-specific associations for each frailty-by-urgency subgroup [18,19]. Stratum-specific estimates were adjusted for age alone because of the limited number of deaths in the sparsest cell. A significance level of α = 0.10 was prespecified for interaction tests, without adjustment for multiple testing across the four analyses. Because several stratum-specific cells contain few deaths, the resulting intervals are wide, and interaction estimates were therefore interpreted as exploratory and descriptive rather than as confirmatory [24]. Confidence intervals for stratum-specific and interaction estimates used cluster-robust standard errors, and those for the RERI used the delta method [25].

Four prespecified sensitivity analyses were conducted: (1) excluding episodes in which postoperative albumin infusion was administered before the landmark time, (2) restricting the analysis to one episode per patient, (3) using an alternative day 3 landmark, and (4) restricting the cohort to patients with complete frailty and comorbidity data. A post hoc analysis restricted the cohort to one episode per inpatient admission (Table S2). No a priori sample size calculation was performed because the cohort size was fixed by the available data window. All analyses were conducted as complete-case analyses without imputation, and statistical tests were two-sided. Analyses added in response to peer review were not prespecified and are labeled post hoc throughout. Analyses were performed in Python 3.12 (Python Software Foundation, Wilmington, DE, USA) using lifelines 0.30.3, scikit-learn 1.6.1, and statsmodels 0.14.6.

## 3. Results

### 3.1. Cohort and Baseline Characteristics

Among the 41,506 eligible surgical episodes, the median age was 69 years, 51.8% were men, 17.0% underwent emergency surgery, and in-hospital mortality was 1.1% (470 deaths) (Table 1). Mortality and baseline-model discrimination at 30, 90, and 365 days in the full cohort are summarized in Table S3; among episodes with a known vital status at 365 days, the mortality rate was 7.0%. Two nested day 7 landmark cohorts were analyzed (Figure 1). Of the 18,296 episodes meeting the postoperative day 7 landmark criterion, 14,992 (81.9%) had a day 7 albumin measurement and formed the landmark Cox cohort (287 in-hospital deaths after the landmark and 1329 deaths within 365 days) and were used for the Cox regression and effect-modification analyses; 14,754 of these with complete baseline covariates formed the discrimination cohort (269 in-hospital deaths after the landmark). The 238 excluded episodes lacked a recorded operative time. Baseline characteristics of both cohorts, including the 52.5% with day 7 hypoalbuminemia, are provided in Table S4. Among the 14,951 landmark episodes with a recorded Major Diagnostic Category, the most frequent were digestive, hepatobiliary and pancreatic surgery (5546; 37.1%), trauma, burns and poisoning (3236; 21.6%), and circulatory surgery (1386; 9.3%); post-landmark in-hospital mortality ranged from 0.00% to 8.52% across the 16 categories (Table S13). Preoperative albumin within 30 days of surgery was available for 13,781 episodes and fell by a median of 0.8 g/dL by day 7 (Table S14). When the two measurements were cross-classified, episodes already hypoalbuminemic before surgery had the highest post-landmark in-hospital mortality (136 of 2166; 6.28%), followed by those that became hypoalbuminemic only after surgery (112 of 5245; 2.14%), and both rates exceeded that among episodes with normal albumin at both time points (25 of 6153; 0.41%) (Table S15).

### 3.2. Prognostic Association and Discrimination

**Figure 2:**
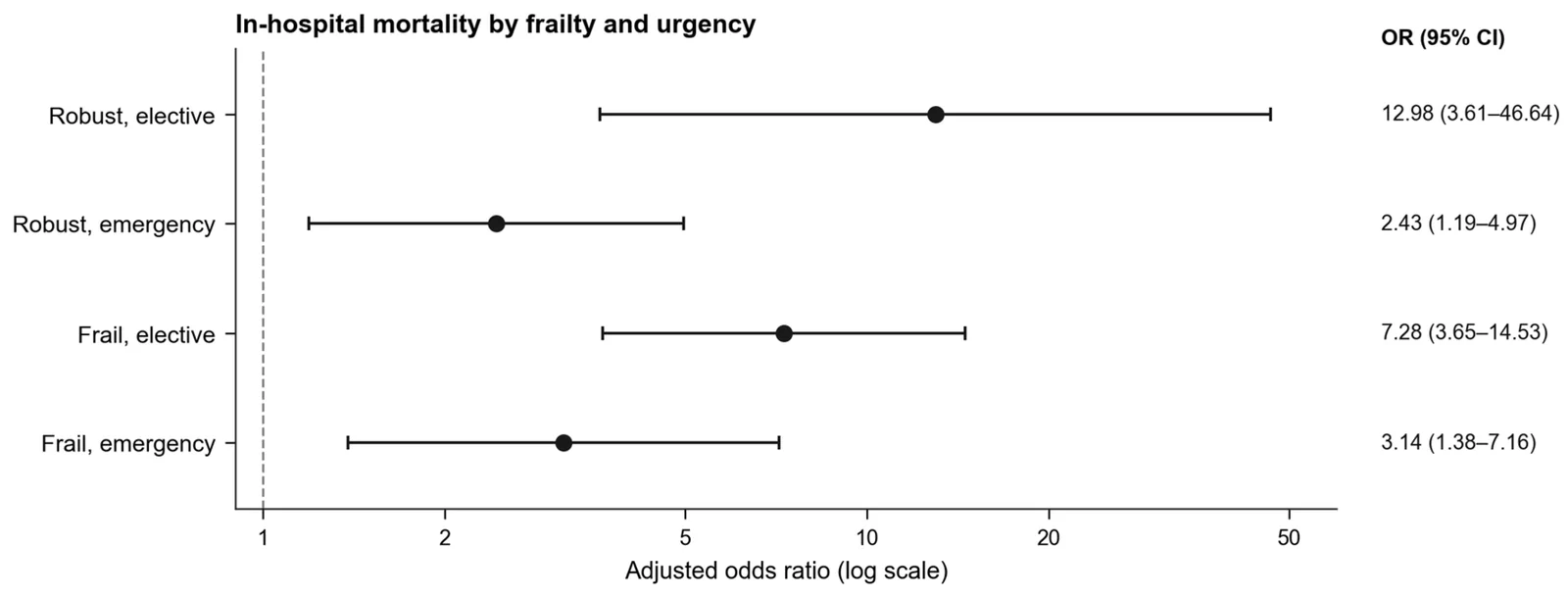
Adjusted odds ratio for in-hospital mortality according to frailty and surgical urgency. Estimates represent the association between day 7 postoperative hypoalbuminemia (albumin < 3.0 g/dL) and in-hospital mortality after the day 7 landmark and were adjusted for age. The strata comprise 7058 episodes with 47 deaths (robust, elective), 2419 with 70 deaths (robust, emergency), 4252 with 107 deaths (frail, elective), and 1263 with 63 deaths (frail, emergency). Markers indicate adjusted odds ratios; horizontal lines represent 95% confidence intervals; and the dashed vertical line indicates the null value. The x-axis is displayed on a logarithmic scale.

Day 7 hypoalbuminemia was associated with 365-day mortality overall (adjusted hazard ratio, 3.43; 95% CI, 2.95–3.98) and within every frailty-by-urgency stratum (Table 2). A similar association was observed for post-landmark in-hospital mortality across all strata, with adjusted odds ratios from 2.43 (95% CI, 1.19–4.97) to 12.98 (95% CI, 3.61–46.64) (Figure 2). The association between day 7 hypoalbuminemia and 365-day mortality violated the proportional hazards assumption (scaled Schoenfeld residuals, *p* < 0.001) and was stronger early in follow-up. In horizon-specific landmark Cox analyses, the adjusted hazard ratio was 6.63 (95% CI, 4.16–10.59) through postoperative day 30 and 4.67 (95% CI, 3.58–6.10) through day 90 (Table S5). The 365-day hazard ratio therefore represents a time-averaged summary of an association that was strongest early. Median follow-up from the landmark was 358 days (interquartile range, 358–358), and 11,245 episodes (75.0%) were followed to the full 365-day horizon.

**Figure 3:**
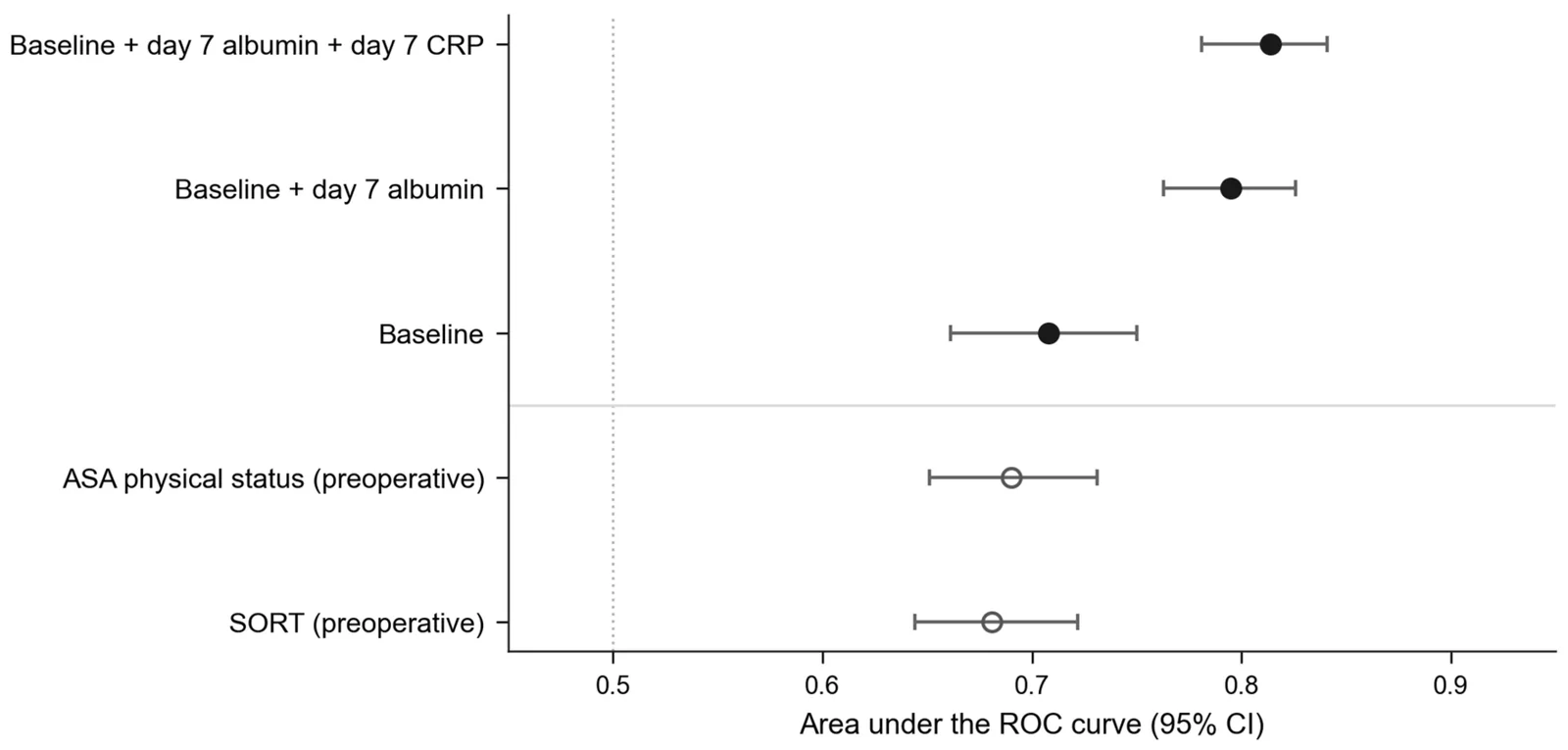
Discrimination of post-landmark in-hospital mortality in a common analytic sample. Discrimination is the area under the receiver operating characteristic curve (95% confidence interval) in a common analytic sample (*n* = 6938; 160 deaths) with complete data for all model variables. Filled markers are the fitted models (baseline, baseline plus day 7 albumin, baseline plus day 7 albumin and day 7 CRP), optimism-corrected by Harrell’s bootstrap. Open markers are the preoperative tools (SORT and ASA physical status) as fixed external scores. Horizontal lines represent 95% confidence intervals, and the dotted vertical line indicates an area under the curve of 0.5. Abbreviations: ASA, American Society of Anesthesiologists; CRP, C-reactive protein; SORT, Surgical Outcome Risk Tool.

Discrimination was assessed in a common analytic sample with both day 7 albumin and C-reactive protein available (11,283 episodes; 258 deaths). The optimism-corrected AUC rose from 0.638 for the routine baseline model (age, surgical urgency, anesthetic type, and operative time) to 0.769 after adding day 7 albumin and to 0.798 after adding day 7 C-reactive protein (Figure 3 and Table S6). In a same-sample head-to-head comparison with complete data for all predictors (6938 episodes; 160 deaths), the day 7 albumin model had a higher AUC than the preoperative tools: AUC was 0.795 (95% CI, 0.763–0.826) for the albumin model and 0.814 (95% CI, 0.781–0.841) with C-reactive protein, versus 0.681 (95% CI, 0.644–0.722) for SORT and 0.690 (95% CI, 0.651–0.731) for ASA physical status (baseline, 0.708). Because comparing models side by side does not establish incremental value, a post hoc nested analysis added day 7 albumin to each preoperative tool within a common complete-case sample (9958 episodes; 175 deaths). The optimism-corrected AUC rose from 0.662 to 0.803 for SORT, from 0.700 to 0.837 for ASA physical status, and from 0.688 to 0.835 for the two combined, with non-overlapping confidence intervals in each pair (Table S16). On temporal internal validation (models developed on 2018–2023 data and validated in a held-out 2024 cohort from the same institution; 1765 episodes; 33 deaths), point estimates were similar (AUC 0.795 for the albumin model and 0.796 with C-reactive protein; baseline, 0.668), but were based on only 33 deaths and represent internal rather than external validation. Apparent calibration differed little between the baseline model and the model with day 7 albumin (Brier score 0.01778 and 0.01727; Table S17), and in decision-curve analysis the albumin model showed greater apparent net benefit across thresholds from 0.001 to 0.196 (Table S18). Both analyses are apparent (in-sample) and do not establish clinical utility.

### 3.3. Interaction with Frailty and Surgical Urgency

No evidence of multiplicative interaction was observed between day 7 hypoalbuminemia and frailty (interaction odds ratio, 0.87; 95% CI, 0.42–1.80; Wald χ^2^(1) = 0.1; *p* = 0.71). The additive interaction was positive but imprecisely estimated (RERI, 2.11; 95% CI, −0.57 to 4.80) (Table 2). The adjusted odds ratio for post-landmark in-hospital mortality was 5.27 for hypoalbuminemia alone, 1.77 for frailty alone, and 8.15 for the combination of hypoalbuminemia and frailty (Table S7). For surgical urgency, the primary analysis showed an exploratory inverse multiplicative interaction (interaction odds ratio, 0.30; 95% CI, 0.13–0.68; Wald χ^2^(1) = 8.3; *p* = 0.004), consistent with a weaker relative association between day 7 hypoalbuminemia and mortality among patients undergoing emergency surgery. The additive interaction was positive but imprecisely estimated (RERI, 6.75; 95% CI, −1.20 to 14.69). The four cells underlying this estimate comprised 5711 episodes with 14 deaths (neither exposure), 5599 with 140 (hypoalbuminemia only), 1404 with 23 (emergency surgery only), and 2278 with 110 (both), so the reference cell is sparse and the interval correspondingly wide. Associations across the three published frailty bands are given in Table S19.

### 3.4. Sensitivity Analyses

All four prespecified sensitivity analyses (excluding episodes receiving pre-landmark albumin infusion, restricting the analysis to one episode per patient, applying a day 3 landmark, and restricting to episodes with complete frailty and comorbidity data) and a post hoc analysis restricted to one episode per inpatient admission yielded results consistent with the primary analysis, with adjusted hazard ratios for 365-day mortality between 3.43 and 3.65 (Table S2). The inverse interaction with emergency surgery was not robust across these sensitivity analyses. The albumin-infusion analysis is not only a robustness check: excluding pre-landmark recipients removed 133 of the 287 in-hospital deaths, changing the population as well as the number of events. In the full surgical cohort, postoperative albumin infusion was given by day 7 in 2462 episodes (5.9%), and recipients had higher in-hospital mortality than non-recipients (7.6% vs. 0.7%; Table S8). Within the landmark cohort, 2435 of 14,992 episodes (16.2%) had received albumin before the landmark. Additionally, adjusting the primary model for Major Diagnostic Category left the association essentially unchanged, with the adjusted hazard ratio for 365-day mortality moving from 3.414 to 3.357 (Table S20). Adjusting instead for the extent of the operation also left it essentially unchanged, moving from 3.32 to 3.25 within the same 14,754 episodes, as did adding the Hospital Frailty Risk Score, which moved it from 3.43 to 3.31 in the full cohort. Adding the preoperative albumin concentration attenuated it substantially, from 3.25 to 1.89 (95% CI, 1.60–2.23) within the same 13,781 episodes, and adding day 7 C-reactive protein attenuated it modestly, from 3.23 to 2.87. With every one of these variables entered together, the adjusted hazard ratio was 1.49 (95% CI, 1.23–1.81), against 2.97 for the primary adjustment set in the same 10,515 episodes (Table S21). When the fall in albumin from the preoperative value was used as the exposure in place of the day 7 threshold, the model yielded an inverse association (hazard ratio 0.62 per 1 g/dL fall; 95% CI, 0.56–0.69) before adjustment for the preoperative value; after adjustment, each 1 g/dL fall was associated with a hazard ratio of 2.22 (95% CI, 1.89–2.61). A large fall can arise only from a high preoperative concentration, so the reversal reflects the mathematical coupling between a change score and its baseline.

## 4. Discussion

In this landmark cohort of patients alive and hospitalized on postoperative day 7, day 7 hypoalbuminemia was associated with an approximately threefold higher hazard of 365-day mortality (adjusted hazard ratio, 3.43), and adding day 7 albumin improved discrimination of post-landmark in-hospital mortality. In a same-sample comparison, the day 7 albumin model had a higher area under the curve than the preoperative SORT and ASA physical status models, and adding day 7 albumin to each of those tools provided additional discriminative information beyond either tool alone; point estimates were similar on temporal internal validation. The association was substantially attenuated, although it persisted, once the preoperative albumin concentration was taken into account. Evidence of effect modification was limited, with no multiplicative interaction with frailty, a positive but imprecise additive interaction, and an exploratory inverse multiplicative interaction with emergency surgery that was not robust across sensitivity analyses.

First, adding day 7 albumin and C-reactive protein improved discrimination, consistent with the possibility that postoperative biomarkers capture the physiological impact of surgery and early complications, which fixed preoperative scores cannot. Both parameters reflect the postoperative inflammatory response rather than separate nutritional and inflammatory processes, and the incremental value of C-reactive protein was smaller on temporal validation than within the development sample. Second, the association with long-term mortality was strongest early in follow-up, so the year-long hazard ratio is a time-averaged effect that may underestimate the early excess risk. Because day 7 values follow surgery and any early complications, they cannot inform preoperative risk stratification, and they are not a substitute for tools measured at a different time for a different purpose. Their potential value lies in the information that only becomes available after surgery, for refining in-hospital surveillance and discharge planning; adding day 7 albumin to SORT or to ASA physical status improved discrimination over either tool alone. If externally validated, these findings may help determine whether a routinely available biomarker can refine in-hospital risk assessment for patients who remain hospitalized after the first postoperative week.

Mechanistically, a low day 7 albumin concentration likely reflects the magnitude and persistence of the postoperative inflammatory response and capillary leak rather than inadequate nutrition alone [9,10], consistent with its stronger association early after surgery and with the reported value of the postoperative C-reactive protein-to-albumin ratio [26,27]. Frailty and low albumin were each associated with mortality [15,28,29,30]; however, their joint association differed little from the sum of their separate effects. Although some preoperative indices incorporate both [16], our findings suggest that their joint association is largely additive rather than synergistic. In the exploratory interaction analysis, the association was attenuated on the multiplicative scale in emergency surgery. Because hypoalbuminemia was more prevalent and baseline mortality higher there, a low value may add less discriminatory information. This may reflect a ceiling effect: when most patients in a group are already at high risk, one further abnormal result shifts the estimate less. The Charlson Comorbidity Index and the Hospital Frailty Risk Score also capture different constructs: chronic comorbidity burden and frailty, so neither substitutes for the other. A single absolute threshold groups patients with different trajectories: those already hypoalbuminemic before surgery had a markedly higher post-landmark mortality than those who became hypoalbuminemic only afterwards.

This study has several limitations. First, and most importantly, the cohort was conditional on being alive and still hospitalized on postoperative day 7, so the findings cannot be generalized to the entire surgical population: they apply to patients whose hospital stay extended beyond 1 week, not to those who died or were discharged earlier. Of the 18,296 landmark-eligible episodes, 82% had a day 7 albumin measurement. Because day 7 testing was not random, the cohort may be enriched for patients requiring closer monitoring. We characterized this selection: compared with landmark-eligible episodes without a day 7 measurement, measured episodes had longer operations, greater comorbidity, and were older, whereas postoperative length of stay, emergency status, and frailty were similar (Table S12). On the measured characteristics, the pattern suggests selection toward sicker surgical patients rather than a healthier subset. That comparison addresses only whether albumin was measured among landmark-eligible episodes. Table S12 does not quantify the earlier selection on survival and continued hospitalization to day 7. Second, the same-sample comparison with SORT and ASA required a documented ASA physical status, available for about three-quarters of episodes under general anesthesia (6938 episodes; 160 deaths), and favors the day 7 model in two respects: the day 7 models were fitted and optimism-corrected within the study cohort, whereas SORT and ASA were developed elsewhere, and prediction was conditional on survival to day 7, which a postoperative biomarker is intrinsically better placed to predict. The observed discrimination gains therefore support incremental prognostic information but do not establish superiority as a calibrated risk model, and both analyses were single-center and require external validation. Third, this observational study cannot support causal inference. Residual confounding by unmeasured severity cannot be excluded, although the associations persisted after adjustment for age, sex, and comorbidity. Adding the preoperative albumin concentration substantially attenuated the estimate, from 3.25 to 1.89 within the same episodes (Table S21), so a substantial part of the association reflects the concentration a patient carried into surgery rather than the postoperative course. The association nonetheless persisted after that adjustment and after all of the additional covariates evaluated in Table S21 were entered together. Procedure type and operative magnitude were not included in the primary adjustment set. In addition, a low day 7 albumin concentration may partly reflect established complications such as anastomotic leak or sepsis, which both reduce albumin and increase mortality. It is therefore best read as an integrative marker of the postoperative course rather than an independent prognostic factor. We cannot separate confounding from mediation in these data, because the dataset records no postoperative complication variables: anastomotic leak, reoperation, surgical site infection, and postoperative sepsis are each absent. A low day 7 albumin, therefore, cannot be decomposed into the part explained by an established complication and the part independent of one. Fourth, the Hospital Frailty Risk Score is claims-based and was developed for acute medical admissions rather than surgical patients. Calibration and decision-curve analysis were assessed only in the sample in which the models were fitted (Tables S17 and S18 and Figure S1), with no external calibration and no formal reclassification analysis, so clinical utility has not been demonstrated. Because discrimination and calibration were assessed for post-landmark in-hospital mortality, we did not treat discharge as a competing event or evaluate incremental discrimination at 365 days. Fifth, albumin infusion may increase measured albumin and is more likely in sicker patients. The sensitivity analysis excluding infusion recipients yielded a similar estimate for the primary association. Because that analysis removed 133 of the 287 in-hospital deaths, the exploratory inverse interaction with emergency surgery did not reach significance there. Residual exposure misclassification cannot be excluded. Finally, the effect-modification analysis was secondary and exploratory, with 287 in-hospital deaths across sparse subgroups. The frailty interactions were not statistically significant, and the inverse multiplicative interaction with emergency surgery (one of four tests, and not robust across the sensitivity analyses) should be interpreted cautiously. Because the odds ratio is non-collapsible, multiplicative estimates are conditional on the adjustment set and not directly comparable with the hazard ratios, so the interaction results are descriptive and scale-dependent. Ascertainment of 365-day vital status was incomplete because patients who did not return to the institution were censored at their last documented contact rather than the end of the observation window, which extended beyond 365 days for every episode; although the death registry captures out-of-hospital deaths reported to the institution, this censoring may nonetheless be informative. The institution holds no linkage to national or municipal death registries, so deaths of patients who did not return could not be ascertained, and a patient who died elsewhere is indistinguishable from one who survived without returning. No a priori sample size calculation was performed, limiting power for interaction.

## 5. Conclusions

Among patients alive and still hospitalized on postoperative day 7, day 7 hypoalbuminemia was associated with higher short- and long-term mortality and provided additional discriminative information when added to routine models and fixed preoperative risk tools, although the association was substantially attenuated once the preoperative albumin concentration was taken into account. Effect modification was limited and exploratory, with no clear interaction with frailty and an inverse interaction with emergency surgery that was not robust, and the association with long-term mortality was strongest early in follow-up. Because day 7 albumin may reflect the overall postoperative course, including early complications, it merits evaluation as a routinely available marker for dynamic postoperative risk stratification, rather than as an independent prognostic factor or a treatment target. This was a single-center observational study with internal validation only; the findings are hypothesis-generating and require external validation of calibration and prospective assessment of clinical utility before clinical use. These findings support prospective evaluation of day 7 albumin, including among frail patients, in whom perioperative optimization improves outcomes [31].

## Figures and Tables

**Table 1:** Baseline characteristics, operative characteristics, and clinical outcomes of the study cohort.

Characteristic	Value
Study episodes (qualifying operations), *n*	41,506
Inpatient admissions, *n*	39,222
Patients, *n*	31,200
*Demographics*	
Age, years, median (IQR)	69 (52–77)
Male, *n* (%)	21,483 (51.8)
Age ≥ 75 years, *n* (%)	13,741 (33.1)
Body mass index, kg/m^2^, median (IQR)	22.7 (20.3–25.5)
*Functional status*	
Admission Barthel index, median (IQR)	20 (17–20)
*Operative characteristics*	
Emergency surgery, *n* (%)	7062 (17.0)
Operative time, min, median (IQR)	76 (45–135)
Anesthesia time, min, median (IQR)	112 (66–193)
Length of hospital stay, days, median (IQR)	8 (4–15)
*Outcomes*	
In-hospital mortality, *n* (%)	470 (1.1)
Discharge home, *n* (%)	36,273 (87.4)
Transfer to another hospital, *n* (%)	3700 (8.9)

Each qualifying operation was treated as a separate episode, so a patient with more than one admission, or an admission with more than one operation, contributes more than one episode. Values are expressed as median (IQR) or *n* (%). Abbreviations: IQR, interquartile range.

**Table 2:** Effect modification of postoperative day 7 hypoalbuminemia on mortality: interaction analyses and stratum-specific effect estimates (secondary, exploratory analyses). (**A**) Interaction with in-hospital mortality. (**B**) Stratum-specific associations with 365-day mortality.

(**A**)			
Effect modifier	Multiplicative OR (95% CI)	*p*-value	Additive RERI (95% CI)
Frailty (HFRS ≥ 5)	0.87 (0.42–1.80)	0.71	2.11 (−0.57 to 4.80)
Emergency surgery	0.30 (0.13–0.68)	0.004	6.75 (−1.20 to 14.69)
(**B**)			
Stratum		Adjusted HR (95% CI)	
Overall (*n* = 14,992; 1329 deaths)		3.43 (2.95–3.98)	
*Robust*			
Elective (*n* = 7058; 438 deaths)		4.21 (3.30–5.37)	
Emergency (*n* = 2419; 172 deaths)		2.42 (1.59–3.69)	
*Frail*			
Elective (*n* = 4252; 532 deaths)		3.10 (2.46–3.90)	
Emergency (*n* = 1263; 187 deaths)		2.44 (1.60–3.72)	

Estimates are presented for in-hospital mortality. Multiplicative interaction is expressed as an interaction odds ratio, whereas additive interaction is expressed as the relative excess risk due to interaction. Interaction estimates were adjusted for age, the Charlson Comorbidity Index, and the other modifier. A significance level of α = 0.10 was prespecified, without adjustment for multiple testing. Estimates are adjusted hazard ratios for 365-day mortality from a landmark Cox model. Episodes and deaths within 365 days are given for each stratum. The overall hazard ratio was adjusted for age, sex, and the Charlson Comorbidity Index, and stratum-specific hazard ratios were adjusted for age. The overall albumin association was non-proportional over 365 days (stronger early), so the hazard ratio is a time-averaged summary. Abbreviations: CI, confidence interval; HFRS, Hospital Frailty Risk Score; RERI, relative excess risk due to interaction; OR, odds ratio; HR, hazard ratio.

## Data Availability

The data presented in this study are available from the corresponding author upon reasonable request. The data are not publicly available because they contain information that could compromise participant privacy and are subject to institutional restrictions governing the sharing of electronic health record data.

## Supplementary Materials

The following supporting information can be downloaded at https://www.mdpi.com/article/10.3390/jcm15197742/s1. Table S1: Exposure, effect modifiers, and adjustment variables; Table S2: Sensitivity analyses; Table S3: Mortality and model discrimination by horizon (full surgical cohort); Table S4: Baseline characteristics of the day 7 landmark cohorts; Table S5: Unadjusted and adjusted association of day 7 hypoalbuminemia with mortality at 30, 90, and 365 days; Table S6: Discrimination of in-hospital mortality after the landmark; Table S7: Four-cell decomposition of the frailty × hypoalbuminemia additive interaction (in-hospital mortality); Table S8: Postoperative albumin administration, by window; Table S9: Distribution of the actual day of the day 7 albumin measurement; Table S10: Association with mortality at an alternative hypoalbuminemia threshold of 3.5 g/dL; Table S11: Reclassification between the 3.0 g/dL and 3.5 g/dL thresholds; Table S12: Characteristics of episodes with and without a day 7 albumin measurement; Table S13: Case mix of the day 7 landmark cohort by Major Diagnostic Category; Table S14: Preoperative albumin and its change to day 7; Table S15: Cross-classification of preoperative and day 7 albumin; Table S16: Nested models adding day 7 albumin to the preoperative risk tools; Table S17: Calibration of the day 7 landmark models; Table S18: Decision-curve analysis of the day 7 landmark models; Table S19: Association by three levels of the Hospital Frailty Risk Score; Table S20: Additional adjustment for the type of surgical procedure; Table S21: Association with 365-day mortality under alternative adjustment sets; Figure S1: Calibration of the day 7 landmark models.

## Abbreviations

The following abbreviations are used in this manuscript:

SORT	Surgical Outcome Risk Tool
ASA	American Society of Anesthesiologists
STROBE	Strengthening the Reporting of Observational Studies in Epidemiology
AUC	Area Under the Receiver Operating Characteristic Curve
CIs	Confidence Intervals
RERI	Relative Excess Risk Due To Interaction

## Appendix A. Supplementary Methods

The main text provides the full methods. Six additional methodological details are provided here.

First, the postoperative day 7 landmark required a postoperative length of stay beyond 7 days, calculated as the interval between surgery and discharge.

Second, the unit of analysis was the surgical episode, not the admission. The 41,506 episodes arose from 39,222 inpatient admissions among 31,200 patients because 1792 admissions (4.6%) included more than one qualifying operation. In the landmark Cox cohort, 14,992 episodes arose from 13,739 admissions among 12,198 patients.

An in-hospital death was an admission-level event; therefore, an admission contributing two episodes counted the same death twice: 162 of the 1329 deaths within 365 days and 57 of the 287 in-hospital deaths after the landmark were such repeat contributions. This dependence was addressed in three ways. Confidence intervals used cluster-robust standard errors on the patient, which was the coarser cluster and, therefore, also absorbed within-admission correlation. Sensitivity analysis S2 restricted the cohort to one episode per patient (adjusted hazard ratio 3.63, 95% CI 3.10–4.26, versus 3.43, 95% CI 2.95–3.98 in the primary analysis). The post hoc analysis S5 directly removed the duplication by restricting the cohort to one episode per inpatient admission, and it reproduced the primary estimate (adjusted hazard ratio 3.45, 95% CI 2.98–4.00; Table S2).

Third, the value transcribed on nutrition care forms was not used to define day 7 albumin because, in patients without a day 7 blood test, it carried the preoperative value forward and would have mixed preoperative values into the “day 7” exposure. The same rule was applied to the alternative day 3 landmark in Table S2, for which the exposure was the laboratory albumin nearest to postoperative day 3 within days 1 to 3, so that the measurement likewise preceded the landmark.

Fourth, the two analytic cohorts were nested. Of the 18,296 episodes meeting the postoperative day 7 landmark criterion, 14,992 (81.9%) had a day 7 laboratory albumin measurement and formed the landmark Cox and effect-modification cohort. Of these, 14,754 with complete baseline covariates formed the discrimination cohort; the 238 excluded episodes were those with a missing operative time. Analyses were complete-case analyses within each cohort.

Fifth, the adjustment sets differed by estimand and are reported with each estimate. The overall association was adjusted for age, sex, and the Charlson Comorbidity Index. Stratum-specific estimates within the four frailty-by-urgency cells were adjusted for age alone, because the limited number of deaths in the sparsest cells did not support a larger prespecified adjustment set. Interaction models were adjusted for age, the Charlson Comorbidity Index, and the other modifier.

Sixth, the relative excess risk due to interaction was defined on the risk-ratio scale. Because in-hospital death after the landmark was uncommon (287 of 14,992 episodes, 1.9%), the odds ratios from the logistic models were used as approximations to relative risks when computing the RERI. The approximation was close at this event rate, but the additive-scale estimates should be interpreted with this limitation in mind.
