# Supplementary material for: Association Between Postoperative Day 7 Hypoalbuminemia and Mortality After Surgery: A Single-Center Retrospective Landmark Cohort Study
Source: J Clin Med. 2026 Oct 8;15(19):7742. doi: 10.3390/jcm15197742 (PMC13665809; doi:10.3390/jcm15197742)

# Supplementary Materials

## Association between postoperative day 7 hypoalbuminemia and mortality after surgery: a single-center retrospective landmark cohort study

## Supplementary Tables

**Table S1. Exposure, effect modifiers, and adjustment variables**

| Role | Variable | Operationalization |
|---|---|---|
| Exposure | Day 7 serum albumin (laboratory) | Laboratory value nearest postoperative day 7 (days 4–7), below 3.0 g/dL (continuous for discrimination) |
| Effect modifier | Frailty | Hospital Frailty Risk Score of 5 or more |
| Effect modifier | Surgical urgency | Emergency versus elective surgery |
| Adjustment | Age | Years |
| Adjustment | Sex | Male or female |
| Adjustment | Comorbidity | Charlson Comorbidity Index |

**Table S2. Sensitivity analyses**

**Panel A. Association of day 7 hypoalbuminemia with mortality across sensitivity analyses**

| Analysis | Episodes | In-hospital deaths | Emergency deaths | Deaths ≤ 365 days | Adjusted HR, 365-day (95% CI) | Adjusted OR, in-hospital (95% CI) |
|---|---|---|---|---|---|---|
| Primary analysis | 14,992 | 287 | 133 | 1,329 | 3.43 (2.95–3.98) | 5.98 (3.95–9.06) |
| S1 Albumin infusion before the landmark excluded | 12,557 | 154 | 54 | 916 | 3.51 (2.96–4.16) | 14.88 (7.84–28.21) |
| S2 One episode per patient | 12,198 | 210 | 114 | 1,013 | 3.63 (3.10–4.26) | 5.78 (3.85–8.66) |
| S3 Alternative day 3 landmark | 23,305 | 331 | 166 | 1,506 | 3.65 (3.22–4.14) | 5.32 (3.94–7.18) |
| S4 Complete frailty and comorbidity data | 14,992 | 287 | 133 | 1,329 | 3.43 (2.95–3.98) | 5.98 (3.95–9.06) |
| S5 One episode per inpatient admission (post hoc) | 13,739 | 230 | 115 | 1,162 | 3.45 (2.98–4.00) | 5.96 (4.04–8.79) |

**Panel B. Interaction with frailty across sensitivity analyses**

| Analysis | Multiplicative interaction OR (95% CI) | *P* | Additive RERI (95% CI) |
|---|---|---|---|
| Primary analysis | 0.87 (0.42–1.80) | .71 | 2.11 (−0.57 to 4.80) |
| S1 | 0.53 (0.14–1.91) | .33 | 14.76 (−3.72 to 33.24) |
| S2 | 0.65 (0.29–1.42) | .28 | 1.57 (−1.16 to 4.29) |
| S3 | 1.56 (0.85–2.87) | .15 | 2.14 (0.39–3.90) |
| S4 | 0.87 (0.42–1.80) | .71 | 2.11 (−0.57 to 4.80) |
| S5 | 0.67 (0.31–1.44) | .30 | 2.12 (−0.68 to 4.91) |

**Panel C. Interaction with emergency surgery across sensitivity analyses**

| Analysis | Multiplicative interaction OR (95% CI) | *P* | Additive RERI (95% CI) |
|---|---|---|---|
| Primary analysis | 0.30 (0.13–0.68) | .004 | 6.75 (−1.20 to 14.69) |
| S1 | 0.57 (0.15–2.07) | .39 | 9.75 (−1.60 to 21.10) |
| S2 | 0.35 (0.15–0.80) | .01 | 8.41 (0.32–16.51) |
| S3 | 0.66 (0.37–1.20) | .17 | 9.27 (3.93–14.61) |
| S4 | 0.30 (0.13–0.68) | .004 | 6.75 (−1.20 to 14.69) |
| S5 | 0.36 (0.16–0.78) | .01 | 8.37 (0.69–16.06) |

The exposure is a day 7 laboratory albumin below 3.0 g/dL (below 3.0 g/dL at the day 3 landmark in S3), and hazard ratios are from the landmark Cox model with censoring at the last documented contact or 365 days from the day of surgery, whichever occurred earlier. Estimation follows the primary analysis exactly: cluster-robust standard errors on the patient, adjustment for age, sex, and the Charlson Comorbidity Index for the overall estimates, and adjustment for age, the Charlson Comorbidity Index, and the other modifier for the interaction estimates, with the RERI interval from the delta method. Panel A also reports in-hospital deaths in the emergency stratum, because the emergency interaction is estimated within it and its event count drives the precision of that estimate. S2 selects, for each patient, the emergency episode where present and otherwise the episode with the longest operating-room time. S5 applies the same rule within each inpatient admission. S5 was added post hoc and was not prespecified. Because an in-hospital death belongs to the admission rather than to the operation, an admission with two qualifying operations contributes that death to two episodes and

carries more weight than an admission with one. S5 removes that duplication directly, leaving one episode per admission, and the primary association is unchanged (adjusted hazard ratio 3.45, 95% CI 2.98–4.00, versus 3.43, 95% CI 2.95–3.98). S4 is identical to the primary analysis because the Hospital Frailty Risk Score and the Charlson Comorbidity Index were complete for every eligible episode. It is retained because it was prespecified. The direction and magnitude of the primary association were stable across the four prespecified perturbations and the post-hoc analysis (adjusted hazard ratio 3.43 to 3.65). The additive interaction estimates were positive in every analysis. The inverse multiplicative interaction with emergency surgery was not robust. It reached conventional significance in the primary analysis and in S2 but not in S1 or S3. The two discordant analyses differ from the primary analysis in ways that explain the loss. S1 excludes the 2,435 episodes that received albumin before the landmark. That removes 133 of the 287 in-hospital deaths overall, and it falls hardest on the stratum the interaction is estimated in. The emergency stratum retains 54 of its 133 deaths. The interaction estimate therefore becomes imprecise rather than reversed (0.57, 95% CI 0.15–2.07, versus 0.30, 95% CI 0.13–0.68). S3 shifts the landmark to day 3, where the exposure is measured before much of the postoperative course has elapsed and the cohort is larger and less selected (23,305 episodes), so the emergency contrast is diluted (0.66, 95% CI 0.37–1.20). Neither analysis reverses the direction. This pattern supports reading the interaction as exploratory. CI, confidence interval; HR, hazard ratio; OR, odds ratio; RERI, relative excess risk due to interaction.

**Table S3. Mortality and model discrimination by horizon (full surgical cohort)**

| Horizon | Observed, n | Deaths, n | Mortality among observed episodes, % | Baseline AUC (95% CI) |
|---|---|---|---|---|
| 30-day | 39,220 | 378 | 1.0 | 0.750 (0.722–0.777) |
| 90-day | 36,249 | 872 | 2.4 | 0.686 (0.670–0.703) |
| 365-day | 32,026 | 2,257 | 7.0 | 0.675 (0.663–0.686) |

This horizon-wise analysis is of the full surgical cohort (N = 41,506), not the day 7 landmark cohort, so the death counts are not comparable with the landmark Cox cohort in the main text (14,992 episodes, 1,329 deaths). “Observed” is the number with confirmed vital status at each horizon (94.5%, 87.3%, and 77.2% at 30, 90, and 365 days). The remainder were censored at their last documented contact with the institution, and mortality is expressed among observed episodes. The baseline model (age, surgical urgency, anesthetic type, and operative time) was fitted on complete cases (n = 38,656, 35,727, and 31,582) and corrected for optimism. AUC, area under the receiver-operating-characteristic curve.

**Table S4. Baseline characteristics of the day 7 landmark cohorts**

| Characteristic | Landmark Cox cohort | Discrimination cohort |
|---|---|---|
| Study episodes, n | 14,992 | 14,754 |
| Inpatient admissions, n | 13,739 | 13,651 |
| Patients, n | 12,198 | 12,126 |
| ***Demographics*** | | |
| Age, years | 73 (63–80) | 73 (63–80) |
| Male, n (%) | 8,194 (54.7) | 8,059 (54.6) |
| Body mass index, kg/m$^2$ | 22.1 (19.6–24.8) | 22.1 (19.6–24.8) |
| Missing body mass index, n (%) | 1,205 (8.0) | 1,173 (8.0) |
| ***Comorbidity and frailty*** | | |
| Charlson Comorbidity Index | 2 (0–4) | 2 (0–4) |
| Charlson Comorbidity Index $\geq$ 3, n (%) | 5,872 (39.2) | 5,762 (39.1) |
| Hospital Frailty Risk Score | 3.2 (0.9–7.0) | 3.2 (0.9–6.9) |
| Hospital Frailty Risk Score $\geq$ 5, n (%) | 5,515 (36.8) | 5,394 (36.6) |
| ***Functional status*** | | |
| Admission Barthel index | 20 (8–20) | 20 (8–20) |
| Missing Barthel index, n (%) | 2,197 (14.7) | 2,143 (14.5) |
| ***Operative*** | | |
| Emergency surgery, n (%) | 3,682 (24.6) | 3,600 (24.4) |
| General anesthesia, n (%) | 11,262 (75.1) | 11,242 (76.2) |
| Operative time, min | 126 (64–207) | 126 (64–207) |
| Postoperative length of stay, days | 14 (11–22) | 14 (11–22) |
| ***Day 7 exposure*** | | |
| Day 7 serum albumin, g/dL | 2.9 (2.5–3.3) | 2.9 (2.5–3.3) |
| Day 7 hypoalbuminemia ($<$ 3.0 g/dL), n (%) | 7,877 (52.5) | 7,711 (52.3) |
| Albumin infusion before the landmark, n (%) | 2,435 (16.2) | 2,384 (16.2) |
| ***Outcome*** | | |
| In-hospital death after the landmark, n (%) | 287 (1.9) | 269 (1.8) |
| Observed death within 365 days of surgery, n (%) | 1,329 (8.9) | 1,279 (8.7) |

Values are median (interquartile range) unless otherwise stated. The two cohorts are nested. The discrimination cohort is the landmark Cox cohort restricted to episodes with complete baseline covariates. Each qualifying operation is a separate episode, so a patient with more than one admission, or an admission with more than one operation, contributes more than one episode. Compared with the full surgical cohort of Table 1, the landmark cohorts are older (median 73 versus 69 years), more often emergency (24.6% versus 17.0%), and have a longer stay, as expected of a cohort restricted to patients whose postoperative course extends beyond a week. Frailty and comorbidity were recorded for every episode, so neither variable contributes missing data. Albumin infusion refers to an infusion given between surgery and the day 7 landmark.

**Table S5. Unadjusted and adjusted association of day 7 hypoalbuminemia with mortality at 30, 90, and 365 days**

| Horizon | Episodes, n | Deaths, n | Unadjusted HR (95% CI) | Adjusted HR (95% CI) |
|---|---|---|---|---|
| 30-day | 14,992 | 182 | 7.00 (4.46–11.01) | 6.63 (4.16–10.59) |
| 90-day | 14,992 | 523 | 5.15 (3.98–6.65) | 4.67 (3.58–6.10) |
| 365-day | 14,992 | 1,329 | 3.91 (3.39–4.50) | 3.43 (2.95–3.98) |

Landmark Cox models in the day 7 landmark cohort, with cluster-robust standard errors on the patient. Unadjusted models contain the exposure alone, whereas adjusted models add age, sex, and the Charlson Comorbidity Index. Both are fitted in the same complete-case set, so the two columns are directly comparable, and adjustment moves the estimate only modestly (for example 3.91 to 3.43 at 365 days). Follow-up starts at the day 7 landmark and each horizon is measured from the day of surgery, with censoring at the earlier of the last documented contact or that horizon. Deaths are therefore those occurring between the landmark and the horizon. The gradient across horizons quantifies the non-proportionality reported in the main text. The association is strongest in the first 30 days and attenuates over the year, so the 365-day hazard ratio is a time-averaged summary that understates the early excess. CI, confidence interval; HR, hazard ratio.

**Table S6. Discrimination of in-hospital mortality after the landmark**

**Panel A. Incremental discrimination on a single common sample (n = 11,283 episodes, 258 deaths)**

| Model | AUC (95% CI) |
|---|---|
| Baseline | 0.638 (0.604–0.672) |
| Baseline + day 7 albumin | 0.769 (0.740–0.797) |
| Baseline + day 7 albumin + day 7 CRP | 0.798 (0.770–0.825) |
| Baseline + day 7 CRP (without albumin) | 0.762 (0.737–0.790) |

**Panel B. Same-sample head-to-head against the preoperative tools (single common sample, n = 6,938 episodes, 160 deaths)**

| Model | Type | AUC (95% CI) |
|---|---|---|
| Baseline | fitted | 0.708 (0.661–0.750) |
| Baseline + day 7 albumin | fitted | 0.795 (0.763–0.826) |
| Baseline + day 7 albumin + day 7 CRP | fitted | 0.814 (0.781–0.841) |
| SORT (Protopapa 2014) | fixed score | 0.681 (0.644–0.722) |
| ASA physical status | fixed score | 0.690 (0.651–0.731) |

**Panel C. Temporal (out-of-time) validation, developed on 2018–2023 and validated on the held-out 2024 (1,765 episodes, 33 deaths)**

| Model | Development AUC | 2024 validation AUC |
|---|---|---|
| Baseline | 0.650 | 0.668 |
| Baseline + day 7 albumin | 0.771 | 0.795 |
| Baseline + day 7 albumin + day 7 CRP | 0.803 | 0.796 |

**Panel D. Optimism correction under episode-level versus patient-level cluster bootstrap (Panel A sample; 11,283 episodes from 9,400 patients, 258 deaths)**

| Model | Apparent AUC | Episode bootstrap | Patient-cluster bootstrap |
|---|---|---|---|
| Baseline | 0.646 | 0.638 | 0.638 |
| Baseline + day 7 albumin | 0.774 | 0.769 | 0.770 |
| Baseline + day 7 albumin + day 7 CRP | 0.803 | 0.798 | 0.799 |

The primary analysis resamples episodes, which are not independent because 11,283 episodes come from 9,400 patients. Panel D repeats the Harrell optimism correction resampling patients with replacement and taking all episodes of each selected patient, which preserves the within-patient dependence. The episode-level column reproduces the Panel A estimates exactly, and the cluster version is lower by at most 0.006, so the reported discrimination is not an artifact of repeated episodes.

The outcome is in-hospital death after the day 7 landmark. The baseline model comprises age, surgical urgency, anesthetic type, and operative time, and all areas under the curve are optimism-corrected (Harrell bootstrap) except the fixed external scores in Panel B (SORT, ASA), which carry bootstrap confidence intervals. Each panel is a single common sample in which every model input is available, so all models are compared in the same episodes. No matching procedure was used. In Panel B, ASA physical status was integrated across all documentation sources (anesthetic preoperative-examination record, anesthetic record, preoperative summary, free-text digest), covering about three-quarters of general-anesthesia cases, and SORT uses the same

integrated ASA. In Panel C, event counts are small (33 deaths), so the validation areas under the curve are imprecise and are reported without confidence intervals. SORT, Surgical Outcome Risk Tool; CRP, C-reactive protein; ASA, American Society of Anesthesiologists; AUC, area under the receiver-operating-characteristic curve.

**Table S7. Four-cell decomposition of the frailty × hypoalbuminemia additive interaction (in-hospital mortality)**

| Cell | Adjusted OR | *n* | In-hospital deaths |
|---|---|---|---|
| Neither (reference) | 1.00 | 5,042 | 19 |
| Hypoalbuminemia alone | 5.27 | 4,435 | 98 |
| Frailty alone | 1.77 | 2,073 | 18 |
| Both | 8.15 | 3,442 | 152 |

Adjusted for age, the Charlson Comorbidity Index, and the other modifier. Hypoalbuminemia is a day 7 albumin below 3.0 g/dL. Frailty is a Hospital Frailty Risk Score of 5 or more. The joint cell (both) exceeds the sum of the single-exposure excesses. The additive interaction is summarized by the relative excess risk due to interaction, RERI 2.11. OR, odds ratio.

**Table S8. Postoperative albumin administration, by window**

| Window | Received, n (%) | Median, g | Mortality, %* |
|---|---|---|---|
| Preoperative | 558 (1.3) | 75 | 11.1 vs 1.0 |
| By day 7 landmark | 2,462 (5.9) | 38 | 7.6 vs 0.7 |
| Any postoperative | 2,637 (6.4) | 50 | 8.4 vs 0.6 |

* Mortality among recipients versus non-recipients. Percentages are of the full surgical cohort (N = 41,506). Within the day 7 landmark cohort, 2,435 of 14,992 episodes (16.2%) had received an infusion before the landmark. Albumin infusion is given preferentially to sicker patients and raises measured albumin. Infused values were not used to define the exposure, and excluding these episodes did not change the association.

**Table S9. Distribution of the actual day of the day 7 albumin measurement**

| Postoperative day | Episodes, n | % of measured | Albumin, g/dL | Below 3.0 g/dL, n | Below 3.0 g/dL, % |
|---|---|---|---|---|---|
| Day 4 | 597 | 4.0 | 2.8 (2.4–3.2) | 333 | 55.8 |
| Day 5 | 1,318 | 8.8 | 2.9 (2.6–3.3) | 664 | 50.4 |
| Day 6 | 2,063 | 13.8 | 2.9 (2.5–3.2) | 1,169 | 56.7 |
| Day 7 | 11,014 | 73.5 | 2.9 (2.5–3.3) | 5,711 | 51.9 |

The exposure is the laboratory albumin nearest postoperative day 7 within days 4 to 7, so that it precedes the landmark. Values are median (interquartile range). Almost three-quarters of measurements (73.5%) were taken on day 7 itself, and the prevalence of hypoalbuminemia varied little across the window (50.4% to 56.7%), so variation in measurement timing is unlikely to drive the reported association. This analysis was added in revision and was post hoc.

**Table S10. Association with mortality at an alternative hypoalbuminemia threshold of 3.5 g/dL**

| Threshold | Below threshold, n (%) | Episodes, n | Deaths, n | Adjusted estimate (95% CI) |
|---|---|---|---|---|
| ***365-day mortality (landmark Cox, hazard ratio)*** | | | | |
| Below 3.0 g/dL (prespecified) | 7,877 (52.5) | 14,992 | 1,329 | 3.43 (2.95–3.98) |
| Below 3.5 g/dL (post hoc) | 12,613 (84.1) | 14,992 | 1,329 | 3.79 (2.81–5.12) |
| ***In-hospital mortality after the landmark (odds ratio)*** | | | | |
| Below 3.0 g/dL (prespecified) | 7,877 (52.5) | 14,992 | 287 | 5.98 (3.95–9.06) |
| Below 3.5 g/dL (post hoc) | 12,613 (84.1) | 14,992 | 287 | 24.41 (5.99–99.43) |

Both thresholds are applied to the same 14,992 episodes, because eligibility depends on whether a day 7 albumin exists and not on its value; only the classification of the exposure changes. Models are those of the primary analysis (age, sex, and the Charlson Comorbidity Index, with cluster-robust standard errors on the patient). The hazard ratio is essentially unchanged, with overlapping intervals, so the association does not depend on where the threshold is placed. The odds ratio at 3.5 g/dL should not be read as a stronger association: raising the threshold leaves only 2,379 unexposed episodes containing 2 in-hospital deaths, and the resulting interval spans a seventeen-fold range. This analysis was added in revision and was post hoc. CI, confidence interval.

**Table S11. Reclassification between the 3.0 g/dL and 3.5 g/dL thresholds**

| Day 7 albumin band | n | % of cohort | In-hospital deaths, n (%) | Deaths $\leq$ 365 days, n (%) |
|---|---|---|---|---|
| Below 3.0 g/dL (exposed at both thresholds) | 7,877 | 52.5 | 250 (3.17) | 1,054 (13.38) |
| 3.0 to below 3.5 g/dL (exposed only at 3.5) | 4,736 | 31.6 | 35 (0.74) | 220 (4.65) |
| 3.5 g/dL or above (unexposed at both) | 2,379 | 15.9 | 2 (0.08) | 55 (2.31) |

Because 3.5 g/dL is a superset of 3.0 g/dL, the two definitions differ only by the middle band. That band carries an in-hospital mortality of 0.74%, between the 3.17% below 3.0 g/dL and the 0.08% at or above 3.5 g/dL, and it accounts for nearly a third of the cohort. Moving the threshold to 3.5 g/dL therefore classifies 84.1% of episodes as abnormal while leaving a reference group too small to estimate against, which is why 3.0 g/dL was retained for the primary analysis. This analysis was added in revision and was post hoc.

**Table S12. Characteristics of episodes with and without a day 7 albumin measurement**

| Characteristic | Measured (n = 14,992) | Not measured (n = 3,304) | SMD |
|---|---|---|---|
| ***Demographics*** | | | |
| Age, years | 73 (63–80) | 68 (53–79) | 0.335 |
| Male, n (%) | 8,194 (54.7) | 1,928 (58.4) | −0.075 |
| Body mass index, kg/m$^2$ | 22.1 (19.6–24.8) | 22.4 (19.8–25.2) | −0.062 |
| ***Comorbidity and frailty*** | | | |
| Charlson Comorbidity Index | 2 (0–4) | 1 (0–3) | 0.385 |
| Hospital Frailty Risk Score | 3.2 (0.9–7.0) | 2.7 (0.0–6.8) | 0.047 |
| Hospital Frailty Risk Score $\geq$ 5, n (%) | 5,515 (36.8) | 1,148 (34.7) | 0.043 |
| ***Preoperative laboratory*** | | | |
| Preoperative albumin, g/dL | 3.8 (3.3–4.2) | 4.0 (3.5–4.4) | −0.223 |
| Preoperative C-reactive protein, mg/dL | 0.29 (0.07–2.32) | 0.18 (0.05–1.12) | 0.212 |
| ***Operative*** | | | |
| Emergency surgery, n (%) | 3,682 (24.6) | 872 (26.4) | −0.042 |
| Operative time, min | 126 (64–207) | 76 (49–125) | 0.429 |
| Postoperative length of stay, days | 14 (11–22) | 14 (9–22) | 0.057 |
| ***Outcome and disposition*** | | | |
| In-hospital death, n (%) | 287 (1.9) | 36 (1.1) | 0.068 |
| Discharge home, n (%) | 10,974 (73.2) | 2,543 (77.0) | −0.087 |
| Transfer to another hospital, n (%) | 2,987 (19.9) | 627 (19.0) | 0.024 |
| Discharge to a long-term care facility, n (%) | 637 (4.2) | 78 (2.4) | 0.106 |

Among the 18,296 episodes meeting the day 7 landmark, 14,992 (81.9%) had a day 7 laboratory albumin and 3,304 (18.1%) did not. Values are median (interquartile range) or n (%). Only characteristics defined independently of the measurement itself are compared; day 7 albumin and day 7 C-reactive protein are excluded because their absence defines the grouping. Day 7 testing was not protocolized, and measured episodes had longer operations, more comorbidity, higher age, and a worse preoperative state. Two comparisons bound how far this selection reaches: postoperative length of stay was identical in the two groups, so measurement does not simply mark a longer stay, and neither prespecified effect modifier was imbalanced (emergency surgery SMD −0.042; Hospital Frailty Risk Score SMD 0.047), so the effect-modification analyses are less exposed to this selection than the overall estimate. Preoperative albumin here uses the institutional default look-back and is not restricted to 30 days. SMD, standardized mean difference; values with an absolute SMD above 0.1 are conventionally regarded as imbalanced. This analysis was added in revision and was post hoc.

**Table S13. Case mix of the day 7 landmark cohort by Major Diagnostic Category**

| Major Diagnostic Category | n | % | In-hospital deaths, n (%) | Day 7 albumin below 3.0 g/dL, % |
|---|---|---|---|---|
| 06 Digestive system, liver, biliary tract and pancreas | 5,546 | 37.1 | 65 (1.17) | 55.0 |
| 16 Trauma, burns and poisoning | 3,236 | 21.6 | 43 (1.33) | 60.1 |
| 05 Circulatory system | 1,386 | 9.3 | 54 (3.90) | 51.8 |
| 11 Kidney, urinary tract and male reproductive system | 1,279 | 8.6 | 8 (0.63) | 36.0 |
| 07 Musculoskeletal system | 1,097 | 7.3 | 12 (1.09) | 43.1 |
| 01 Nervous system | 766 | 5.1 | 36 (4.70) | 49.2 |
| 04 Respiratory system | 428 | 2.9 | 23 (5.37) | 55.8 |
| 12 Female reproductive system, puerperium and abnormal pregnancy | 353 | 2.4 | 8 (2.27) | 58.1 |
| 18 Other | 247 | 1.7 | 11 (4.45) | 59.1 |
| 10 Endocrine, nutritional and metabolic | 205 | 1.4 | 5 (2.44) | 20.0 |
| 13 Blood, haematopoietic organs and immune system | 176 | 1.2 | 15 (8.52) | 53.4 |
| 03 Ear, nose, mouth and throat | 141 | 0.9 | 6 (4.26) | 56.7 |
| 08 Skin and subcutaneous tissue | 51 | 0.3 | 0 (0.00) | 51.0 |
| 14 Neonatal and congenital anomalies | 23 | 0.2 | 0 (0.00) | 17.4 |
| 09 Breast | 12 | 0.1 | 0 (0.00) | 41.7 |
| 02 Eye | 5 | 0.0 | 0 (0.00) | 0.0 |

Categories are Major Diagnostic Categories of the Japanese Diagnosis Procedure Combination system, ordered by size. The denominator is the 14,951 episodes of the day 7 landmark cohort with a recorded category; 41 episodes (0.3%) had none and are excluded. Percentages of episodes are of that denominator, and mortality percentages are within each category. The cohort is dominated by abdominal and trauma surgery, and in-hospital mortality after the landmark varies more than sevenfold across categories, from 0.63% in urological surgery to 8.52% in haematological disease, which is the case mix against which the day 7 albumin finding should be generalized. This analysis was added in revision and was post hoc.

**Table S14. Preoperative albumin and its change to day 7**

| Preoperative window | With a pre-operative value, n (%) | Deaths, n | Preoperative albumin, g/dL | Day 7 albumin, g/dL | Absolute change, g/dL | Relative change, % |
|---|---|---|---|---|---|---|
| Within 30 days (primary) | 13,781 (91.9) | 285 | 3.8 (3.2–4.2) | 2.9 | −0.8 (−1.2 to −0.4) | −21.1 |
| Within 7 days (sensitivity) | 9,188 (61.3) | 264 | 3.6 (3.0–4.0) | 2.8 | −0.7 (−1.1 to −0.3) | −19.4 |

Contrary to the assumption that a baseline value is unavailable, a preoperative albumin exists for most of the day 7 landmark cohort. Values are median (interquartile range) and percentages are of the 14,992 episodes in that cohort. The institutional laboratory feed permits a look-back of up to one year, so the window was narrowed for this analysis; it could be narrowed but not widened. Albumin fell by a median of 0.8 g/dL, or about a fifth of the preoperative value, between surgery and day 7. Preoperative hypoalbuminemia (below 3.0 g/dL) was already present in 2,383 episodes (17.3%) within the 30-day window and 2,237 (24.3%) within the 7-day window, the difference reflecting that patients measured close to surgery are a sicker subset. This analysis was added in revision and was post hoc.

**Table S15. Cross-classification of preoperative and day 7 albumin**

| Preoperative → day 7 | n | In-hospital deaths, n | Mortality, % | Median change, g/dL |
|---|---|---|---|---|
| Normal → normal | 6,153 | 25 | 0.41 | −0.7 |
| Normal → low (postoperative decline) | 5,245 | 112 | 2.14 | −1.1 |
| Low → normal | 217 | 12 | 5.53 | +0.5 |
| Low → low (already hypoalbuminemic) | 2,166 | 136 | 6.28 | −0.1 |

Normal is an albumin of 3.0 g/dL or above and low is below 3.0 g/dL, applied to the preoperative value within 30 days of surgery and to the day 7 value, in the 13,781 episodes with both. An absolute day 7 threshold does group two different situations together, as the reviewer notes, and they do not carry the same risk: patients who were already hypoalbuminemic before surgery had an in-hospital mortality of 6.28%, roughly three times the 2.14% of those who fell below the threshold only after surgery. The small low-to-normal cell is the group whose albumin rose, and it retains an intermediate mortality. This analysis was added in revision and was post hoc.

**Table S16. Nested models adding day 7 albumin to the preoperative risk tools**

| Model | Optimism-corrected AUC (95% CI) |
|---|---|
| SORT alone | 0.662 (0.620–0.702) |
| SORT + day 7 albumin | 0.803 (0.774–0.830) |
| ASA physical status alone | 0.700 (0.649–0.739) |
| ASA physical status + day 7 albumin | 0.837 (0.814–0.864) |
| SORT + ASA physical status | 0.688 (0.635–0.730) |
| SORT + ASA physical status + day 7 albumin | 0.835 (0.812–0.861) |

All six models are fitted in a single common sample of 9,958 episodes containing 175 in-hospital deaths after the landmark, so they are directly comparable. The outcome is in-hospital death after the day 7 landmark and all areas under the curve carry Harrell bootstrap optimism correction. The sample is smaller than the landmark cohort because SORT returns no value when ASA physical status is undocumented. Adding day 7 albumin raised discrimination over each preoperative tool alone, and the confidence intervals of each nested pair do not overlap. Combining both preoperative tools did not exceed either tool plus albumin, so the gain comes from the postoperative measurement rather than from adding predictors. This sample is larger than the 6,938 episodes of the earlier same-sample head-to-head comparison, which additionally requires a day 7 C-reactive protein; requiring it here would shrink the sample without serving the nested comparison, which does not use it. SORT, Surgical Outcome Risk Tool; ASA, American Society of Anesthesiologists; AUC, area under the receiver-operating-characteristic curve; CI, confidence interval. This analysis was added in revision and was post hoc.

**Table S17. Calibration of the day 7 landmark models**

**Panel A. Summary**

| Model | Brier score | Hosmer–Lemeshow $\chi^2$ | df | *P* |
|---|---|---|---|---|
| Baseline | 0.0178 | 8.42 | 8 | .39 |
| Baseline + day 7 albumin | 0.0173 | 12.12 | 8 | .15 |

**Panel B. Observed and expected in-hospital mortality by decile of predicted risk**

| | Baseline | | | Baseline + day 7 albumin | | |
|---|---|---|---|---|---|---|
| Decile | n | Deaths | Obs / Exp, % | n | Deaths | Obs / Exp, % |
| 1 | 1,476 | 8 | 0.54 / 0.76 | 1,476 | 1 | 0.07 / 0.16 |
| 2 | 1,477 | 12 | 0.81 / 0.97 | 1,475 | 3 | 0.20 / 0.29 |
| 3 | 1,473 | 18 | 1.22 / 1.08 | 1,475 | 4 | 0.27 / 0.43 |
| 4 | 1,476 | 14 | 0.95 / 1.17 | 1,476 | 5 | 0.34 / 0.61 |
| 5 | 1,475 | 15 | 1.02 / 1.27 | 1,475 | 11 | 0.75 / 0.83 |
| 6 | 1,475 | 22 | 1.49 / 1.40 | 1,475 | 22 | 1.49 / 1.12 |
| 7 | 1,478 | 34 | 2.30 / 1.65 | 1,476 | 28 | 1.90 / 1.54 |
| 8 | 1,473 | 29 | 1.97 / 2.09 | 1,475 | 30 | 2.03 / 2.17 |
| 9 | 1,475 | 53 | 3.59 / 3.12 | 1,475 | 62 | 4.20 / 3.31 |
| 10 | 1,476 | 64 | 4.34 / 4.66 | 1,476 | 103 | 6.98 / 7.79 |

The outcome is in-hospital death after the day 7 landmark in 14,754 episodes containing 269 deaths. Deciles are of predicted risk, and "Obs / Exp" gives the observed mortality followed by the mean predicted risk in that decile. Neither model showed evidence of miscalibration. Two caveats govern how these figures should be read. The discrimination models standardize every predictor and are L2-penalized, which shrinks predictions toward the mean, and the calibration reported here is apparent rather than optimism-corrected, unlike the areas under the curve elsewhere in this supplement. The Hosmer–Lemeshow statistic is reported with its degrees of freedom because the number of deciles determines them. This analysis was added in revision and was post hoc.

**Table S18. Decision-curve analysis of the day 7 landmark models**

| Threshold probability | Baseline + day 7 albumin | Baseline | Treat all |
|---:|---:|---:|---:|
| 0.011 | 0.01117 | 0.00804 | 0.00731 |
| 0.031 | 0.00504 | 0.00194 | −0.01318 |
| 0.051 | 0.00244 | −0.00060 | −0.03453 |
| 0.101 | 0.00066 | 0.00000 | −0.09207 |
| 0.151 | 0.00041 | 0.00000 | −0.15638 |
| 0.196 | 0.00035 | 0.00000 | −0.22110 |

Values are net benefit for in-hospital death after the day 7 landmark, evaluated on a grid of threshold probabilities from 0.001 to 0.196 in steps of 0.005; six representative thresholds are shown. Treating no one has a net benefit of zero at every threshold. The model including day 7 albumin exceeded both default strategies at all 40 grid points, whereas the baseline model did so from 0.006 upward, and it exceeded the baseline model throughout. At a threshold of 0.031, for example, its net benefit was 0.00504 against 0.00194 for the baseline model and −0.01318 for treating all. Like the calibration estimates, these curves are apparent rather than optimism-corrected. This analysis was added in revision and was post hoc.

**Table S19. Association by three levels of the Hospital Frailty Risk Score**

| Stratum | Score | n | Deaths | Adjusted OR (95% CI) |
|---|---|---|---|---|
| Overall | — | 14,992 | 287 | 5.98 (3.95–9.06) |
| ***Three levels*** | | | | |
| Low risk | $< 5$ | 9,477 | 117 | 5.51 (3.05–9.98) |
| Intermediate risk | 5 to 15 | 4,693 | 138 | 6.38 (3.57–11.41) |
| High risk | $> 15$ | 822 | 32 | 2.57 (0.79–8.34) |
| ***Dichotomy used in the main analysis*** | | | | |
| Robust | $< 5$ | 9,477 | 117 | 5.51 (3.05–9.98) |
| Frail | $\geq 5$ | 5,515 | 170 | 5.49 (3.27–9.22) |

The outcome is in-hospital death after the day 7 landmark, and every estimate in this table is adjusted for age, sex, and the Charlson Comorbidity Index, as for the overall estimate, with cluster-robust standard errors on the patient. Bands follow Gilbert et al., in which a score of exactly 15 falls in the intermediate group. The three-level split does not change what the dichotomy shows: the two binary strata are almost identical, and the apparent difference between the intermediate and high bands rests on 32 deaths in 822 episodes with an interval that includes the null, so it is too imprecise to support a conclusion of weaker association. Because the adjustment set here matches the overall estimate, whereas the stratum-specific estimates reported in the main text are adjusted for age alone, the two sets of values are not directly comparable. OR, odds ratio; CI, confidence interval. This analysis was added in revision and was post hoc.

**Table S20. Additional adjustment for the type of surgical procedure**

**Panel A. Association with 365-day mortality**

| Model | n | Deaths | Adjusted HR (95% CI) |
|---|---|---|---|
| Age, sex, and Charlson Comorbidity Index | 14,951 | 1,328 | 3.41 (2.94–3.96) |
| + Major Diagnostic Category | 14,951 | 1,328 | 3.36 (2.89–3.90) |

**Panel B. Discrimination of in-hospital mortality after the landmark**

| Model | n | Deaths | Optimism-corrected AUC (95% CI) |
|---|---|---|---|
| Baseline + day 7 albumin | 14,714 | 269 | 0.795 (0.773–0.817) |
| + Major Diagnostic Category | 14,714 | 269 | 0.829 (0.808–0.854) |

The primary model adjusts for age, sex, and the Charlson Comorbidity Index, and so for neither the extent nor the type of the operation; the extent is addressed separately in Table S21. Panel A adds the Major Diagnostic Category as a set of indicator variables to the primary landmark Cox model, in the 14,951 episodes with a recorded category, and the association with day 7 hypoalbuminemia is essentially unchanged. Panel B shows that surgical category nonetheless carries independent prognostic information for in-hospital mortality. These two results answer different questions and should not be conflated: the type of procedure improves prediction, but it does not account for the albumin association. Category was entered without collapsing sparse levels, because the Cox model estimates no stratum-specific effects and a sparse indicator does not destabilize the coefficient of interest. HR, hazard ratio; AUC, area under the receiver-operating-characteristic curve; CI, confidence interval. This analysis was added in revision and was post hoc.

**Table S21. Association with 365-day mortality under alternative adjustment sets**

**Panel A. Day 7 hypoalbuminemia as the exposure**

| Model | n | Deaths | Adjusted HR (95% CI) |
|---|---|---|---|
| ***Extent of the operation*** | | | |
| Age, sex, and Charlson Comorbidity Index | 14,754 | 1,279 | 3.32 (2.86–3.86) |
| + operative time and emergency status | 14,754 | 1,279 | 3.25 (2.79–3.78) |
| ***Preoperative albumin*** | | | |
| Age, sex, and Charlson Comorbidity Index | 13,781 | 1,274 | 3.25 (2.79–3.78) |
| + preoperative albumin | 13,781 | 1,274 | 1.89 (1.60–2.23) |
| ***Postoperative inflammation*** | | | |
| Age, sex, and Charlson Comorbidity Index | 11,495 | 1,095 | 3.23 (2.72–3.84) |
| + day 7 C-reactive protein | 11,495 | 1,095 | 2.87 (2.40–3.43) |
| ***Frailty*** | | | |
| Age, sex, and Charlson Comorbidity Index | 14,992 | 1,329 | 3.43 (2.95–3.98) |
| + Hospital Frailty Risk Score | 14,992 | 1,329 | 3.31 (2.85–3.85) |
| ***Every variable above, entered together*** | | | |
| Age, sex, and Charlson Comorbidity Index | 10,515 | 1,017 | 2.97 (2.49–3.53) |
| + all of the above | 10,515 | 1,017 | 1.49 (1.23–1.81) |

**Panel B. The fall in albumin from before surgery to day 7 as the exposure**

| Model | n | Deaths | Adjusted HR per 1 g/dL fall (95% CI) |
|---|---|---|---|
| Age, sex, and Charlson Comorbidity Index | 13,781 | 1,274 | 0.62 (0.56–0.69) |
| + preoperative albumin | 13,781 | 1,274 | 2.22 (1.89–2.61) |

The outcome is death within 365 days, estimated by landmark Cox regression with cluster-robust standard errors on the patient, as in the primary analysis. Within each block of Panel A the two rows are fitted in exactly the same episodes, so the difference between them is the effect of the adjustment and not of a change in sample. The blocks differ from one another in size because each is confined to the episodes that carry the variable being added. Adjusting for the extent of the operation, and separately for frailty, leaves the association essentially unchanged. Adjusting for preoperative albumin attenuates it substantially, which indicates that a large part of the association reflects the concentration a patient carried into surgery rather than the postoperative course. The association persists after that adjustment and after entering all of the additional covariates above together. C-reactive protein is reported here as a sensitivity analysis and not as a candidate for the primary model, because the postoperative inflammatory response lies on the path between the operation and a low day 7 albumin, so adjusting for it removes part of the association under study. The Hospital Frailty Risk Score is prespecified as an effect modifier, and moving it into the adjustment set changes the estimand. It is shown here only to demonstrate that the placement does not drive the result. In Panel B the fall in albumin appears protective until the preoperative value is held constant, because a large fall can arise only from a high preoperative concentration. The reversal is a consequence of the mathematical coupling between a change score and its baseline, and it is why the absolute day 7 concentration is reported alongside the change rather than the change alone. HR, hazard ratio; CI, confidence interval. This analysis was added in revision and was post hoc.

## Supplementary Figures

**Figure S1. Calibration of the day 7 landmark models.**

Observed mortality plotted against the mean predicted risk within each decile of predicted risk, for the routine baseline model and for the same model with day 7 albumin added, in the 14,754 episodes of the discrimination cohort containing 269 in-hospital deaths after the day 7 landmark. The dotted line is the line of perfect calibration. Points below it indicate that the model predicted more deaths than occurred, and points above it that it predicted fewer. Both models track the diagonal closely across the range of predicted risk, and neither shows evidence of systematic miscalibration. The numerical values, with the Brier score and the Hosmer–Lemeshow statistic, are in Table S17. Two caveats govern how the figure should be read. The models standardize every predictor and are L2-penalized, which shrinks predictions toward the mean, and the calibration shown is apparent rather than optimism-corrected, unlike the areas under the curve reported elsewhere in this supplement. This analysis was added in revision and was post hoc.

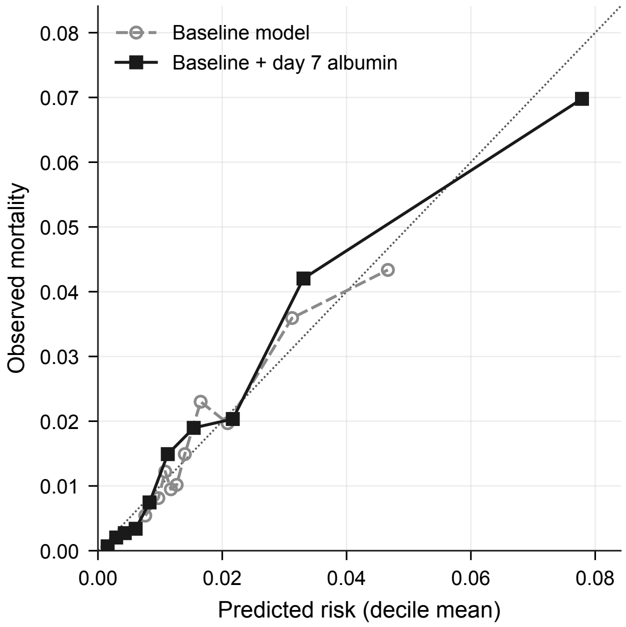

Supplement: Supplementary file 1 [file jcm-15-07742-s001.zip › jcm-4568825-supplementary.pdf]
